# Effective Transfer Learning with Label-Based Discriminative Feature Learning

**DOI:** 10.3390/s22052025

**Published:** 2022-03-04

**Authors:** Gyunyeop Kim, Sangwoo Kang

**Affiliations:** School of Computing, Gachon University, Seongnam 13120, Korea; gyop0817@gachon.ac.kr

**Keywords:** natural language processing, transfer learning, pre-training, word embedding

## Abstract

The performance of natural language processing with a transfer learning methodology has improved by applying pre-training language models to downstream tasks with a large number of general data. However, because the data used in pre-training are irrelevant to the downstream tasks, a problem occurs in that it learns general features rather than those features specific to the downstream tasks. In this paper, a novel learning method is proposed for embedding pre-trained models to learn specific features of such tasks. The proposed method learns the label features of downstream tasks through contrast learning using label embedding and sampled data pairs. To demonstrate the performance of the proposed method, we conducted experiments on sentence classification datasets and evaluated whether the features of the downstream tasks have been learned through a PCA and a clustering of the embeddings.

## 1. Introduction

Artificial intelligence has shown good performance through deep learning from large numbers of data. According to [1], transfer learning conducted through pre-learning with large numbers of data can improve the performance of downstream tasks. Transfer learning refers to pre-learning with unsupervised data that is easy to collect. We proceed with the learning of downstream tasks using a pre-learning model. These processes demonstrate the advantage of an easy collection of unsupervised datasets, which can improve the performance of a downstream task. Therefore, many current artificial intelligence methods use transfer-learning models to achieve a high performance.

In natural language processing (NLP), transfer learning has shown significant performance improvements when applied to language models. In NLP, transfer-learning-based language models such as BERT [2] and ELECTRA [3] are pre-learned using large numbers of natural language data that have been crawled, such as Wiki datasets. Because the data built through crawling make up an unsupervised dataset, learning progresses through semi-supervised learning, such as a masked token preparation. This pre-learned language model is used as a model for generating word embeddings during fine tuning. During the fine-tuning process, downstream task learning is conducted through the construction of a model, including a pre-learning model.

However, the pre-learning model applied in transfer learning uses a dataset that is independent of the downstream task. Thus, during the pre-training process, the model learns general features rather than features specific to downstream tasks. Word embeddings derived through the pre-trained model may have a higher percentage of common features than the information required for downstream tasks. As a result, word embeddings derived from pre-trained models can have unnecessary features in downstream tasks. Furthermore, fine-tuning using the word embeddings through a pre-trained model can be compromised by the unnecessary features presented in the word embeddings.

In this study, further learning is applied to induce pre-trained models to derive word embeddings optimized for downstream tasks. Using the proposed method, word embeddings derived from the pre-learning model allow the features of the downstream tasks to be learned and aim to improve the performance in such tasks. The experimental code of this study can be found at https://github.com/KimGyunYeop/Effective-transfer-learning-with-label-based-discriminative-feature-learning (accessed on 7 February 2022).

## 2. Background

In this paper, we propose a novel method for improving the problem of word embedding that is derived from natural language processing (NLP) using transfer learning. Therefore, this section describes embeddings using transfer learning.

The NLP dataset consists of characters and embedding is used to transform the data into a vector form, thereby enabling them to be used as learning data. Advances in embedding methodologies have resulted in the production of vectors, which include the contextual information of words in sentences. Since the probabilistic n-gram model, i.e., bag-of-words [4] was first developed, methods using deep learning have been proposed and further advances have been made, including ELMo [5] and BERT, whereby contextual information is included in the word information to derive contextual embeddings and transformers.

Previous embedding methodologies have applied the same method to vector words as the bag-of-words [4] approach. Subsequently, studies have been conducted to incorporate the meaning of a word into the vector, and as a result, the method of adding semantic representations over a natural network was proposed [6]. Following this, word2vec [7], which is a method for efficiently learning word embeddings through negative sampling, was proposed, and it exhibited good performance. Word2vec is learned based on the probability of the simultaneous appearance of two words, such that each word is mapped to a vector with a real meaning, and the distance between the vectors is represented as the similarity between the two words. Word2vec can be divided into Skip-gram [7] and CBOW [8], based on the differences in the learning methods. Glove [9] and FastText [5], which use co-occurrence throughout a sentence, have subsequently been proposed.

A problem with the previous methods is the non-contextual results of the embedding. Therefore, to solve this problem, a method for including contextual information in word embedding, namely ELMo, was proposed. ELMo attempts to solve words with different meanings depending on the context, even if they are the same words. The composition of a language model using bidirectional LSTM enables different embedding results to be derived using co-occurrence words. A transformer was later proposed, and a BERT approach using masked token preparation for the transformer and transfer learning was developed, exhibiting good performance in many NLP tasks. Advanced methodologies such as RoBERTa [10], ALBERT [11], and ELECTRA [3] have also been developed. Until recently, the methodology of using language models for word embedding has achieved the highest performance in many NLP tasks.

## 3. Related Work

With the development of transformer and transfer learning, a language model was proposed and good performance was derived. Transformer-based language models (such as BERT [2] and RoBERTa [10]) have been developed by the proposal of several new methods in the pre-training process. Most of the proposed pre-training methodologies perform pre-training through general and large datasets, and learn with a “loss function” that is unrelated to the downstream tasks such as masked token prediction and next sentence prediction. Therefore, the pre-training and fine-tuning processes in the downstream tasks often differ in many language models. Several studies have presented methodologies to narrow the gap between the pre-training and fine-tuning that occurs at this stage. DialoGPT dialoGPT [12] and dialogBERT [13] are methods that can pre-train through an interactive dataset rather than a general dataset, such as a conventional wiki dataset. In Splinter [14], pre-training is performed by adding a recurring span selection for learning in a form that is similar to QA. As such, methodologies for reducing the gap between the pre-training and fine-tuning have been steadily developed. In [15], a method was proposed to narrow the difference in the domain between the data of the pre-training and downstream tasks, and the required information. Pre-training using wiki datasets may lead to a lack of information on the target area for learning through general data that are unrelated to the downstream tasks, which may adversely affect the fine-tuning. To solve this problem, a loss function that induces the narrowing of the sentence embedding distance between data of the same label during the fine-tuning process was proposed in [15]. Through the loss function, sentence embedding that is specialized for downstream tasks is induced to be learned. This paper presents a method to solve the problem of prior learning that was proposed in [15]. Existing methods may face problems whereby the sentence embedding may be adjusted in different directions for each sample. This problem is described in detail in Section 4.3. In this study, to overcome this challenge, we propose a method for intensively learning features that are related to downstream tasks, rather than general features that are learned in pre-training during fine-tuning.

## 4. Proposed Method

The method proposed in this paper aims to induce word embeddings of pre-learned models to obtain more suitable results for downstream tasks. The proposed method discriminates the data using label information shown in the downstream dataset. Thus, each word embedding is changed to encourage distinct embedding.

**Figure 1:**
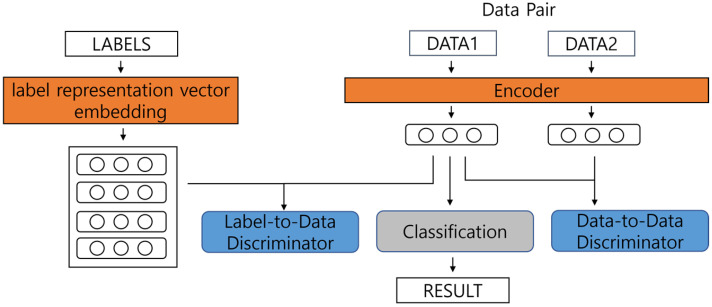
Overall architecture of proposed framework with classification.

Figure 1 illustrates the entire process used by the proposed method. As shown in Figure 1, the proposed methodology is learned through three methods: the main process, label-to-data discrimination, and data-to-data discrimination. Section 4.1 describes the main process used when employing the existing learning methods. In Section 4.2, we compare the two data against the data-to-data crime process, which compares two data based on the agreement of the labels proposed in [15]. Section 4.3 describes problems that can arise from the methodologies consisting only of Section 4.1 and Section 4.2. Section 4.4 describes the label-to-data discriminate process for constructing label embeddings to solve the problem described in Section 4.3.

The loss function of proposed method is given by Equation (1):

(1) $$\begin{array}{c} L=L_{m}+d_{1}L_{dd}+d_{2}L_{ld} \end{array}$$

which combines the loss function of the main process $L_{m}$, the loss function $L_{dd}$ of Equation (6), and the loss function $L_{ld}$ of Equation (8), as described in Section 4.3. $d_{1}$ and $d_{2}$ are the hyperparameters for adjusting the ratio of each loss.

### 4.1. Main Process

The main process is the process of learning downstream tasks. In this study, we constructed and used a simple model for document classification in the experiments. Therefore, in the main process of this paper, we proceed with document classification. The constructed model derives word embeddings through data encoders, as shown in Figure 1. Subsequently, we classify the sentence embedding (i.e., the result of the [CLS] token of BERT) $v\in R^{d}$ the encoder results and predict the output $p\in R^{\left|C\right|}$ of the probability for each label. *C* is the set of possible labels, |C| represents the number of elements in the possible labels set, and d is the number of sentence embedding dimensions of the data.

(2) $$\begin{array}{c} p=softmax(Wv+b) \end{array}$$

(3) $$\begin{array}{c} L_{m}=crossentropyloss\left(p\right) \end{array}$$

Equation (2) represents the feedforward network for deriving the final classification result *p*. $W\in R^{|C|\times d}$ and $b\in R^{\left|C\right|}$ are the learnable parameters of the feedforward natural network. Equation (3) is a loss function for learning the main process.

### 4.2. Data-to-Data Discriminate Process

The data-to-data discovery process uses the same learning process as that proposed in [15]. This section is the process of learning by comparing the labels of two randomly sampled pairs. In this process, the goal is to optimize a pre-learning model to extract features suitable for downstream datasets. The proposed method learns comparisons between the labels of downstream tasks. This process induces the learning of the sentence embedding, which is optimized for downstream tasks. In the experiment presented in this paper, it is assumed that the randomly extracted batch is a sampling set. Subsequently, all data pairs inside the batch are used as sampling data pairs. That is, training dataset *D* samples using the sampled dataset *S*, $D\subset \left\{S_{1},S_{2},\ldots S_{n}\right\}$, where *n* is the number of sampled sets. The sampled dataset *S* includes the sentence embedding of all data, $S_{n}\subset \left\{v_{1}^{n},v_{2}^{n},\ldots ,v_{N_{n}}^{n}\right\}$. $N_{n}$, where $N_{n}$ is the number of data in sampled set $S_{n}$. We compare all possible sentence embedding pairs of set $S_{n}$. We compare all possible sentence embedding pairs of the set $S_{n}$. To compare the two datasets, the computation was conducted using the cosine similarity. If the two data have the same label, the loss function induces the cosine similarity to increase and make the same feature. If the labels of the two data are different, the loss function induces the cosine similarity to decrease, leading to different features. By using the cosine similarity value of the two-sentence embedding as a loss, we optimize the pre-learning model in the downstream task.

(4) $$\begin{array}{c} CS\left(a,b\right)=\frac{ab}{∥a∥∥b∥} \end{array}$$

(5) $$\begin{array}{c} D\left(a,b\right)=\left\{\begin{array}{ccc} 1-CS(a,b) & if & Y_{a}=Y_{b}\\ max(0,CS(a,b)-m) & if & Y_{a}\neq Y_{b} \end{array}\right. \end{array}$$

(6) $$\begin{array}{c} L_{dd}=\sum_{s=0}^{n}\sum_{i=0}^{N_{s}}\sum_{j=0}^{N_{s}}D\left(v_{i}^{s},v_{j}^{s}\right) \end{array}$$

The proposed method of the discriminator is shown in Equations (4)–(6). Equation (4) compares data *a* and *b* by calculating the cosine similarity of the two datasets. Equation (5) compares the two data according to the label consistency. Equation (6) learns the direction in which the cosine similarity is increased when the labels of both data are the same for all sampling data and toward the direction in which the cosine similarity is decreased when the labels of the two data are different. The proposed method configures the corresponding loss to compare the two data and progresses the learning such that the sentence embedding of each data is distant or close. This also leads to information regarding each label being contained in the sentence embeddings.In Equations (5) and (6), $Y_{a}$ and $Y_{b}$ represent the label of each *a* and *b*, respectively. Therefore, Equations (5) and (6) induce learning to increase the cosine simplicity if the label of the data pair is the same and to decrease it if the label is different. *m* is a hyperparameter for specifying the range of the cosine similarity, *n* represents the number of sampling sets, $N_{s}$ represents the number of data in the *s*-th sample. $v_{i}^{s}$ and $v_{j}^{s}$ are the sentence embedding of the *i*-th and *j*-th data in *s*-th sample, respectively.

### 4.3. Problems in Sections Data-to-Data Discriminate Process

The pre-trained model is not optimized for downstream tasks. The pre-trained model learns the general features such as grammar and context. Therefore, the embedding results of the pre-learning model do not have sufficient features distinguishing the labels of downstream task. Thus, in a pre-trained model, the data of the same label can have different features. In other words, the pre-trained language model mainly learns the general features of the sentences and fewer features for each label. As a problem with the above case, data with the same label do not have consistent features. If we only use Section 4.2 in the pre-trained model, the model optimizes different directions each time. Section 4.2 method makes the distance between the two embeddings close or distant depending on the label, because in the embedding result of the pre-learning model there is less difference, according to the label. However, this method has a problem, as the direction of learning is different in each sample.

For example, in the first sample for sentiment classification learning, it is assumed that positive data exist primarily in the positive x-dimensional direction, and negative data exist primarily in the negative direction. However, in the next sample, positive data may exist in the negative direction and negative data may exist in the positive direction, which occurs because the pre-learning process learns the general features of the text and not the label information. Therefore, in the case of a data-to-data discriminate process that is learned through a data comparison, all sampling data may have a different direction for learning. In addition, as a worst case, all vectors can converge at one point.

In this study, the proposed method aims at transforming a sentence embedding into an encourage distinct embedding, which clearly distinguishes each data. However, if the learning direction is different for each sampling data, it does not have a consistent learning direction during the construction process, thereby encouraging distinct embedding, and thus, does not learn label-separated encouraging of distinct embedding based on the label, which may adversely affect the final performance assessment. In this study, a clustering-based experiment was conducted to determine whether the proposed problem affects the performance, as outlined in Section 5.4.

### 4.4. Label-to-Data Discriminate Process

To address the problems presented in Section 4.3, we add a new learning process in this study.

The proposed method adds a label-to-data discrimination process to solve the above problems. The label-to-data discrimination process uses label embeddings that can be used for all sampling data. Label embeddings are learnable parameters and generate an identical label embedding for each label. Using label embeddings, data from the same label are always guided to be learned in the same direction. In the label-to-data discrimination process, the label embeddings are learned by comparing the label embeddings with the sentence embeddings that have the label. The loss function measures the cosine similarity between the two vectors and learns the direction in which the cosine similarity increases. This induces sentence embedding to learn the features of the label embedding. The equation of $D_{ld}$ for the training loss of the label-to-data discrimination process is as follows:

(7) $$\begin{array}{c} D_{ld}\left(a,b\right)=\left\{\begin{array}{ccc} 1-CS(a,b) & if & Y_{a}=Y_{b}\\ 0 & if & Y_{a}\neq Y_{b} \end{array}\right. \end{array}$$

(8) $$\begin{array}{c} L_{ld}=\sum_{s=0}^{n}\sum_{i=0}^{N_{s}}\sum_{j=0}^{\left|C\right|}D_{ld}\left(v_{i}^{s},c_{j}^{le}\right) \end{array}$$

Through Equations (7) and (8), label embeddings and the corresponding statements of the data measure the cosine similarity of the embeddings and use the values as loss functions. In this formula, CS is the cosine similarity, as shown in (4). In Equations (7) and (8), *n* represents the number of sampling sets and $v_{i}^{s}$ represents the sentence embedding of the *i*-th data in *s*-th sample. Finally, |C| represents the number of elements in the set of possible labels, whereas $c_{j}^{le}$ is the label embedding of the *j*-th label and consists of a learnable parameter.

## 5. Experiment

In this section, a performance evaluation conducted to demonstrate the performance of the proposed model is described. We compared the performance with that of the existing model in the text classification task. For the experiment, a simple text-classification model was constructed for easy comparison. For classification, the model obtains the sentence embedding of each data and uses a single-layer feed-forward network to obtain the probability of each classification label.

### 5.1. Dataset

We experimented using several sentence classification datasets to confirm the sentence classification performance of the proposed model. For the experiment, MR, SST-2, and SST-5 provided by the Senteval [16] dataset were used for an emotional analysis. In addition, learning was conducted using the news classification datasets R8 [17] and 20news [18]. Both datasets classify the following information into sentences:

MR—Sentiment is classified into positive or negative sentiment polarities of movie reviews [16].
R8—Text of Reuters newswire documents is classified into eight categories [17].
20news—The 20 newsgroups dataset comprises approximately 18,000 newsgroups posts on 20 topics [18].
SST-2—Binary sentiment classification of movie reviews is applied [16].
SST-5—Multi-class sentiment classification of movie reviews is used. The labels are positive, somewhat positive, neutral, somewhat negative, and negative [16].

Table 1 shows the statistics of each dataset and information on the dataset.

### 5.2. Experiment Setup

We compared the performance of the existing pre-trained model with that of the proposed method. The BERT-base [2] and ELECTRA-base [3] models were used as baselines to prove their application in several pre-trained language models. The proposed method was also learned using the same encoder as the baseline. The hyperparameters for each experiment are listed in Table 2. The average of the three experiments was used for all accuracy scores to improve the reliability of the experiment. The experiment was divided into AM and ANN, which was the same as the method classified in [15]. In the process of constructing $d_{dd}$, AM applied the loss function only when the two labels were the same (Equation (5)), and ANN applied the loss function only when the two labels were different (Equation (5)).

### 5.3. Experiment Result

Table 3 shows the experiment results, demonstrating the performance of the proposed method using BERT as an encoder. TThe proposed model showed a good performance for most sentence classifications. In the MR dataset, the validation and test data have values of 84.7 and 85.6. respectively, showing performance improvements of 0.5 and 0.6 in comparison to [11], which is the basis of the baseline and proposed methods. The 20news dataset also showed a performance of 68.9 for the proposed (ANN) method, which is an improvement of 0.3 compared to previous studies. For SST-5, the evaluation and test data were 50.7/52.4, showing performance improvements of 0.7 and 0.5 compared to the previous approaches. However, in the case of the SST-2 dataset, the performance was rather lowered in the AM case, but the performance was improved in the ANN. In addition, the R8 dataset exhibited the highest performance when using the proposed method.

Table 4 and Table 5 shows the experiment results of the proposed method using ELECTRA and RoBERTa as an encoder. Overall, it showed the same tendency as the experiment results of the proposed model using BERT. All test cases showed a performance improvement for the proposed model, and some validation data showed a higher performance for the method by [15]. The possible reason for this phenomenon is described in Section 4.4.

We measured the *p*-value for each dataset and case to verify whether the proposed method actually improved the performance. The results are presented in Table 6. First, compared to the baseline and proposed model, ANN exhibited a high performance improvement in the *p*-value of 0.05, and in the case of AM, both AM and ANN exhibited a statistical significance of 0.1 or less when the proposed method was applied to the existing method. Thus, it was verified that ANN produces better performance than AM in general, and when the proposed method was applied, higher significance of the performance improvement was demonstrated.

### 5.4. Analysis of Results

**Figure 2:**
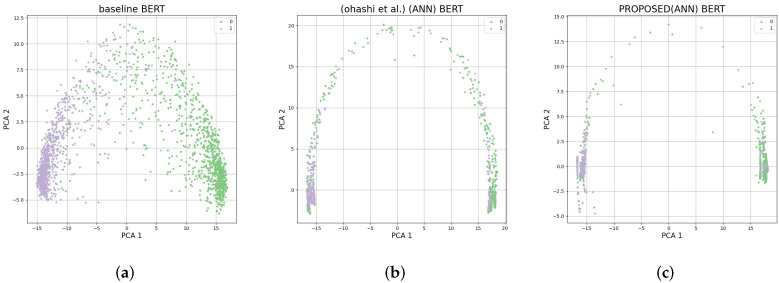
PCA projection of SST-2 test data’s sentence embedding.

The features of the downstream data are learned during fine-tuning. Therefore, if the sentence embedding learning is performed effectively on the target field in binary classification, data with the same labels should be better clustered. In this study, an additional experiment was conducted to confirm the effect of the proposed method on the sentence embedding. All sentence embeddings of the test data of binary classification dataset SST-2 were visualized through principal component analysis (PCA). In this manner, it could be confirmed whether the data for each label were well clustered or whether there were several outliers. The results are presented in Figure 2. When the proposed method was used, as indicated in Figure 2, it was confirmed that better clustering was achieved than when using previous approaches or the baseline, and with fewer outliers. Moreover, to confirm this quantitatively, K-means clustering was performed on the sentence embedding and classification was subsequently applied for each class. Thereafter, a performance evaluation was conducted by applying the completeness score to the clustering results, the results of which are displayed in Table 7. This experiment was conducted on SST-2 and MR, which are binary classification datasets. The baseline achieved clustering scores of 23.4 and 27.4 in both datasets. When the method of [15] was applied, the SST-2 data exhibited high accuracy but the MR data exhibited very low accuracy. It can be observed that the problem described in Section 4.3 occurred. In Section 4.3, we presented a problem, where in the worst case, all sentiment embeddings regardless of the label are clustered in one point, making it difficult to distinguish. In the MR dataset, it was confirmed that classification through clustering became difficult when the method of [15] was applied. When using the proposed method, most of the results showed a higher clustering performance than prior approaches. However, in some cases, the clustering performance improved by a small margin or showed a higher performance at the baseline. The method proposed in this paper uses a methodology for preparing for the worst-case scenario in which the directionality is lost during the data learning applied through the data sampling process. Thus, some cases in the original model may be optimized in the right direction, and in this case, the performance may not be improved. This can be assumed to be the reason for the small performance improvement or the slight decline in performance shown in Table 3, Table 4 and Table 5. However, when the proposed method is used, a low clustering performance is not derived, preventing the occurrence of the worst-case scenario.

## 6. Conclusions

In this study, a methodology has been proposed to compensate for the difference between pre-training and downstream tasks in transfer learning. In this paper, we first described existing research to solve the problem. Among these, a loss function that narrows the distance of sentiment embedding as an existing methodology for solving text classification has been described in detail. Thereafter, we explained that the learning direction cannot be consistent for each sample, due to a problem that may occur when the methodology is used. Finally, label embedding was proposed to solve the problem. The problem was solved by configuring a vector that penetrates all samples through the proposed label embedding. Subsequently, the performance was confirmed through experiments and clustering for text classification. The proposed methodology has solved some of the problems, but exhibits a limitation in that it may not be able to learn effectively, because it does not actually have information on the label. If the initial labeling is incorrectly declared, it is possible that the learning direction of the labeling will not be the same. Future studies can improve the methodology by developing a label embedding initialization method to overcome the limitations. Furthermore, in this study, although the difference in the features required by pre-training and fine-tuning was compensated for, other methods can be proposed to compensate for this gap.

## Figures and Tables

**Table 1:** Description of data.

Dataset	|C|	Number of Training Data	Number of Validation Data	Number of Test Data	Avg. Vocab	Max. Vocab
MR	2	6398	2132	2132	22	6
R8	8	5485	-	2189	103	965
20news	2	6532	-	2568	110	1040
SST-2	2	67,349	872	1821	11	57
SST-5	5	8544	1101	2210	19	56

**Table 2:** Hyperparameter setting in experiment.

Hyperparameter	Value
Sample size (batch size)	256
Max vocab length	50
Max vocab length (R8, 20news)	100
Epoch	30
Learning rate	2 × 10${}^{-5}$
Optimizer	Adam
*m*	−0.75
$d_{1}$	0.5
$d_{2}$	0.5

**Table 3:** Model performance on text classification with BERT.

Model	MR		Test	R8	20news	SST-2		Test	SST-5		Test
	Validation					Validation			Validation		
Baseline	83.9		85.4	97.6	68.7	91.6		92.9	50.0		52.2
[15] (AM)	84.1		85.1	97.7	67.7	91.6		93.2	48.5		49.8
[15] (ANN)	84.2		85.0	97.6	68.6	91.8		92.7	50.0		51.9
Proposed (AM)	84.6		85.9	97.8	68.4	91.6		92.7	50.8		51.4
Proposed (ANN)	84.7		85.6	97.8	68.9	91.9		93.2	50.7		52.4

**Table 4:** Model performance on text classification with ELECTRA.

Model	MR		Test	R8	20news	SST-2		Test	SST-5		Test
	Validation					Validation			Validation		
Baseline	90.0		89.8	97.6	66.9	94.9		95.2	54.1		57.2
[15] (AM)	90.0		89.4	97.6	67.5	93.9		94.5	53.7		57.5
[15] (ANN)	90.3		89.0	97.7	67.7	94.9		95.4	52.4		54.9
Proposed (AM)	89.7		89.6	97.7	67.3	94.8		95.2	53.1		57.6
Proposed (ANN)	90.2		90.1	97.8	67.6	95.1		95.5	54.1		57.7

**Table 5:** Model performance on text classification with RoBERTa.

Model	MR		Test	R8	20news	SST-2		Test	SST-5		Test
	Validation					Validation			Validation		
Baseline	86.4		88.4	97.7	68.2	93.3		95.2	52.8		55.2
[15] (AM)	86.3		88.5	97.6	67.8	93.3		95.5	52.4		55.2
[15] (ANN)	86.8		87.6	97.5	68.1	93.3		95.4	52.9		56.0
Proposed (AM)	88.0		88.6	97.9	68.2	94.3		95.1	53.7		56.1
Proposed (ANN)	88.0		88.4	97.8	68.4	94.0		95.6	54.5		56.3

**Table 6:** *p*-value between the proposed model and the baseline, or [15].

Criteria	Propose	MR		Test	R8	20news	SST-2		Test	SST-5		Test	Avg
Model	Model	Val					Val			Val			
Baseline	AM	0.08		0.19	9.5e-06	0.43	0.23		0.24	0.16		0.35	0.21
Baseline	ANN	0.18		0.10	0.01	0.01	0.14		0.29	0.03		0.01	0.05
[15] (AM)	AM	0.02		0.15	0.004	0.16	0.03		0.01	0.01		0.08	0.10
[15] (ANN)	ANN	0.07		0.004	0.02	0.09	0.09		0.08	0.001		0.08	0.04

**Table 7:** K-means clustering with PCA in binary classification.

DATASET	MODEL	BERT	ELECTRA	RoBERTa
MR	Baseline	23.4	10.0	28.7
	[15] (ANN)	2.6	0.2	27.9
	Proposed(ANN)	43.3	28.7	27.4
SST-2	Baseline	27.4	67.5	67.9
	[15] (ANN)	59.2	10.0	67.0
	Proposed(ANN)	60.1	59.5	69.1

## Data Availability

MR, SST-2, and SST-5 can be found in https://github.com/facebookresearch/SentEval (accessed on 7 February 2022). R8, 20news can be found in https://ana.cachopo.org/datasets-for-single-label-text-categorization (accessed on 7 February 2022).
